# An UWB Physical Optics Approach for Fresnel-Zone RCS Measurements on a Complex Target at Non-Normal Incidence

**DOI:** 10.3390/s19245454

**Published:** 2019-12-11

**Authors:** Ilie Valentin Mihai, Razvan Tamas, Ala Sharaiha

**Affiliations:** 1 IETR (Institute of Electronics and Telecommunications of Rennes), Univ Rennes, UR1, UMR CNRS 6164, F-35000 Rennes, France; ala.sharaiha@univ-rennes1.fr; 2 Doctoral School of Electronics, Telecommunications and Information Technology, University Politehnica of Bucharest, Bucharest 061071, Romania; 3 Department of Electronics and Telecommunications, Constanta Maritime University, Constanta 900663, Romania; tamas@ieee.org

**Keywords:** fresnel region, radar cross section, non-normal incidence, rectangular plate, complex target

## Abstract

In this paper, we propose a fast method for measuring the radar cross section of a complex target at non-normal incidences and Fresnel region antenna-to-target distances. The proposed method relies both on the physical optics approach and on averaging the field distribution over the transmitting and receiving antenna apertures. The ratio between the analytical expression of the radar cross section at far-field and Fresnel region results in a field-zone extrapolation factor. The RCS resulting from the scattering parameters measured at Fresnel region distances is then corrected with that field-zone extrapolation factor. The method is suitable to be used in a perturbed, multipath environment by applying the distance averaging technique, coupling subtraction or time gating. Our technique requires a very simple measuring configuration consisting of two horn antennas and a vector network analyzer. The experimental validation of the proposed technique demonstrates reasonable agreement with simulated radar cross section at non-normal incidence.

## 1. Introduction

Radar cross section (RCS) measurements are generally performed in anechoic chambers or in an open area test site (OATS), under far-field conditions. Large targets, such as aircrafts, vessels, and other large vehicles either cannot be placed inside an anechoic chamber, or the cost of such a measuring site would be prohibitive. Conversely, RCS measurements at far-field ranges in an OATS are faced to ground reflections and a low signal-to-noise ratio (SNR). Moreover, classical near-field-to-far-field transformations are complex, time-consuming, and expensive to be implemented at a large scale, and therefore not suitable for processing data in a real-time scenario. In that case, techniques for RCS evaluation from measurements at Fresnel region radar-to-target distances in a perturbed, multipath environment might be needed. The radar cross section of large, complex targets can also be evaluated on small scale models [1,2,3,4,5,6,7,8]. The far-field, radar cross section of a simplified model consisting of rectangular patches and slots can be computed analytically; it may therefore serve as a reference for comparison purposes when extracting the RCS by processing Fresnel-zone data.

There are several simple methods to approximate the radar cross section (RCS) of different targets at Fresnel region ranges, based on the geometric optics (GO) or physical optics (PO). Geometric optics is an approximate approach based on ray-tracing from the radar to the specular points on the surface of the target [9]. The method fails when evaluating the RCS of flat targets, as the radius of curvature is infinite. Physical optics overcomes this inconvenient by approximating the Stratton–Chu integral equation for the scattered field. The current density $J_{s}$ induced on the surface of a flat or nearly flat perfect electric target is found by assuming the tangent plane approximation:

(1) $$J_{S}=\left\{\begin{array}{cc} 2n\times H_{i}, & \mathrm{in}\;\mathrm{the}\;\mathrm{specular}\;\mathrm{region}\\ 0, & \mathrm{in}\;\mathrm{the}\;\mathrm{shadowed}\;\mathrm{region} \end{array}\right.$$

where n is the normal unit vector at the surface of the target and $H_{i}$ is the tangential component of the incident magnetic field. An advantage of the PO is the ease to find the scattered field by integrating the current distribution on the surface of the target; conversely, the approximation is only valid at normal or near normal incidence $\left(\theta \leq 20^{\circ }\right)$ [10,11,12,13].

The phase deviation between the contributions of different source points to the scattered field should be calculated, in order to find the RCS of a target using PO. Such an evaluation is more difficult in the Fresnel region due to the reactive components of the field and the complexity of the surface integrals to be computed. Several approaches have been proposed, in order to overcome these shortcomings when computing the phase deviation at short distances through modified Green’s functions [14,15,16] and approximations in the phase term [17,18,19,20].

For a radar-to-target distance falling in the near-field zone the field scattered by a dielectric slab can be found through a PO surface integral on equivalent current distributions by using exact Green’s functions [14]; a more accurate representation of the Green’s function led to an improved form for the near-field PO RCS for some complex targets [15]. A similar expression of the phase term in Fresnel region was derived in [16], by performing an expansion in an arbitrary point instead of the origin. In [17], the PO integral is adapted to Fresnel region scattering by phase approximations and surface partitioning. A similar stationary phase method is used in [18] to determine the field distribution near an electrically large conductor. The field scattered by a rectangular, metallic plate in the Fresnel region can be found from the PO surface integral computed by using the adaptive Gauss–Lobatto integration method [19]. The RCS of perfectly-conducting flat targets such as rectangular plates or disks can be evaluated at oblique incidence and below the lower limit of the Fraunhofer range by using the PO approach [20]. The deviation in the phase term can be approximated through Fresnel integrals in order to reduce the complexity and consequently, the computing time. An approach based on a Fresnel region to far-field transformation is presented in [21]; the RCS of a metal plate is evaluated and a simplified aircraft model is characterized. The scattering problem on a metallic, rectangular target in the Fresnel region is analyzed in [22] by using the Helmholtz-–Kirchhoff formula and Babinet’s principle. Horn antennas are generally preferred for RCS measurements in the Fresnel and near-field regions [23]. In a previous work [24], we proposed a method to measure the RCS of a rectangular target at Fresnel zone ranges and normal incidence, in a real environment and a narrow band by using low-directivity antennas, such as Vivaldi or log–periodic antennas. In that case, the strong mutual coupling between antennas and the impedance mismatch impact on the accuracy of the RCS evaluated in the Fresnel region. Due to a quite low signal-to-coupling ratio along with a low antenna gain our previous method only allows RCS measurements at normal incidence (θ=0). Other RCS measuring techniques at short distances were proposed in [25,26,27,28,29,30,31,32,33,34,35,36,37], by taking the PO into consideration.

Most of the methods above apply PO at short antenna-to-target distances, but only for narrow-band analysis on basic target shapes; such techniques may also require a complex measuring configuration. Data processing, including the evaluation of phase deviation usually results in a long computing time, which makes it difficult to apply such a method to a real-time scenario. However, in many practical cases an ultra-wide band (UWB) analysis on complex shape targets may be needed. In this paper, we present a fast UWB technique for measuring the RCS of a complex target at non-normal incidences and Fresnel region antenna-to-target distances. The technique consists of computing a field-zone extrapolation factor to be applied on Fresnel zone RCS measured data, in order to extrapolate the results to the far-field zone. The field-zone extrapolation factor was derived as the ratio between theoretical RCS figures computed in the far-field and Fresnel zones. Moreover, compared to the methods presented before, the technique for measuring the RCS in the Fresnel region proposed in this article requires a very simple measuring configuration consisting of two antennas and a vector network analyzer (VNA). Additionally, the method is suitable to be used in a perturbed multipath environment by applying the distance averaging technique [38], coupling subtraction or time gating. The phase deviation in the PO surface integral was expressed in terms of Fresnel integrals; thus, the computing time was reduced from a couple of hours to a few minutes. We validated our technique by analyzing a simplified small-scale model of camping car side. In this case, the RCS analysis was performed by considering $\phi =0^{\circ }$ and varying θ between $0^{\circ }$ and $20^{\circ }$. The results can be extended by varying the ϕ angle as well provided that the tangent plane approximation in Equation (1) was applied with respect to the electric field.

The paper is organized as follows: a theoretical approach for evaluating the RCS in the Fresnel and far-field zones is firstly presented and an analytical field-zone extrapolation factor is derived; a computing time saving technique with Fresnel integrals is then developed and experimental results are eventually provided.

## 2. Analytical Evaluation of the RCS

### 2.1. Case of a Rectangular Plate

**Figure 1:**
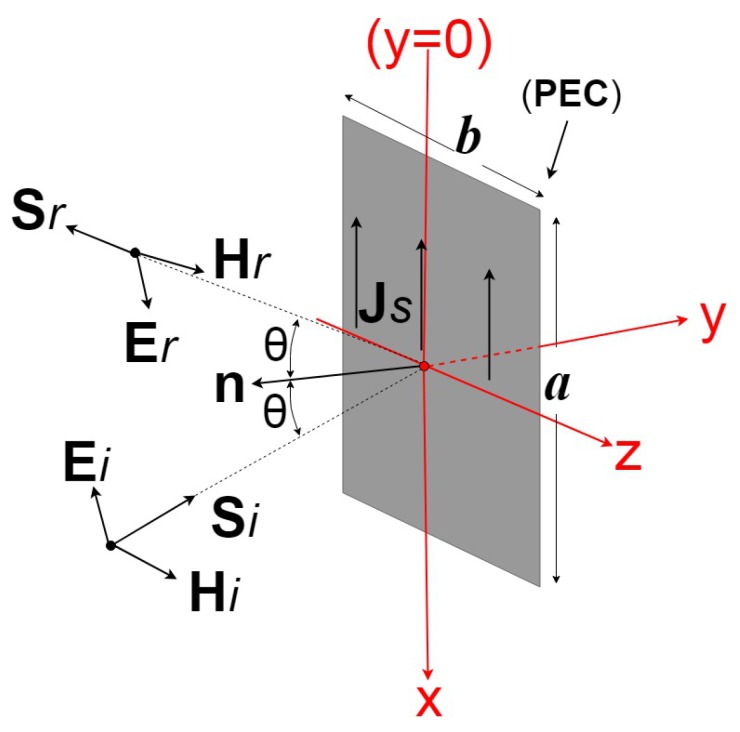
Reflected plane wave at non-normal incidence on a rectangular plate.

The magnetic field $H_{r}$ reflected by a plate of size *a* by *b* (Figure 1) at a distance *d* can be expressed as follows:

(2) $$\begin{array}{c} H_{r}=j\frac{k\exp(-jkd)}{4\pi d}\cos\theta \int_{-\frac{b}{2}}^{\frac{b}{2}}\int_{-\frac{a}{2}}^{\frac{a}{2}}J_{s}\exp\left(-jk\Delta r\right)dx^{\prime }dz^{\prime }. \end{array}$$

**Figure 2:**
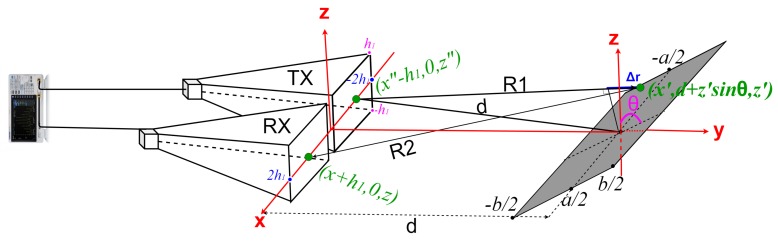
Measuring setup with horn antennas and a rectangular plate.

In Equation (2), $J_{S}$ is the current distribution induced by an incident magnetic field $H_{i}$ on the surface of the plate, *k* is the wave number, *d* is the distance between the antennas and the target, θ is the incidence angle, and Δr is the path length difference between any point on the target of coordinates $\left(x^{\prime },d,z^{\prime }\right)$ and the reference point in the middle of the target (Figure 2).

By cumulatively considering:

- the tangent plane approximation in a specular region ($J_{S}=2n\times H_{i}$),
- a distance *d* falling within the Fresnel zone,
- a single source point on the transmitting antenna $\left(x^{\prime \prime }-h_{1},0,z^{\prime \prime }\right)$,
- a single receiving point on the receiving antenna $\left(x+h_{1},0,z\right)$,

The RCS at ranges in the Fresnel region can be defined as

(3) $$\begin{array}{c} \sigma_{Fr}\left(z^{\prime \prime },z\right)=4\pi d^{2}{\left|\frac{H_{r}}{H_{i}}\right|}^{2}=\frac{4\pi \cos^{2}\theta }{\lambda^{2}}{\left|\int_{-\frac{b}{2}}^{\frac{b}{2}}\int_{-\frac{a}{2}}^{\frac{a}{2}}\exp\left(-jk\Delta r\right)dx^{\prime }dz^{\prime }\right|}^{2}. \end{array}$$

In practice, an infinite number of source points and field points on the transmitting and receiving antenna apertures participate to the radar link. By expressing Δr as in Appendix A and by averaging the field distribution over the apertures of both antennas, the RCS at ranges in the Fresnel region is finally found as:

(4) $$\begin{array}{c} \begin{array}{cc} \; & \sigma_{Fr}=\frac{4\pi \cos^{2}\theta }{\lambda^{2}}|\frac{1}{{\left(2h_{1}\right)}^{4}}\int_{-h_{1}}^{h_{1}}\int_{-h_{1}}^{h_{1}}\int_{-h_{1}}^{h_{1}}\int_{-h_{1}}^{h_{1}}\int_{-\frac{b}{2}}^{\frac{b}{2}}\int_{-\frac{a}{2}}^{\frac{a}{2}}\exp[-jk(\frac{{\left(z-z^{\prime }\right)}^{2}}{2d}+\frac{{\left(z^{\prime \prime }-z^{\prime }\right)}^{2}}{2d}+\frac{{\left(z^{\prime }\right)}^{2}}{2d}\\ \; & +\frac{{\left(x^{\prime }-\left(x^{\prime \prime }-h_{1}\right)\right)}^{2}}{2d}+\frac{{\left(x^{\prime }-\left(x+h_{1}\right)\right)}^{2}}{2d}+2z^{\prime }\sin\theta )]dx^{\prime }dz^{\prime }dzdz^{\prime \prime }dxdx^{\prime \prime }{|}^{2}=\frac{4\pi \cos^{2}\theta }{\lambda^{2}}Q_{Fr_{plate}}^{2} \end{array} \end{array}$$

where

(5) $$\begin{array}{c} \begin{array}{cc} Q_{Fr_{plate}}^{2} & =|\frac{1}{{\left(2h_{1}\right)}^{4}}\int_{-h_{1}}^{h_{1}}\int_{-h_{1}}^{h_{1}}\int_{-h_{1}}^{h_{1}}\int_{-h_{1}}^{h_{1}}\int_{-\frac{b}{2}}^{\frac{b}{2}}\int_{-\frac{a}{2}}^{\frac{a}{2}}\exp[-jk(\frac{{\left(z-z^{\prime }\right)}^{2}}{2d}+\frac{{\left(z^{\prime \prime }-z^{\prime }\right)}^{2}}{2d}+\frac{{\left(z^{\prime }\right)}^{2}}{2d}\\ \; & +\frac{{\left(x^{\prime }-\left(x^{\prime \prime }-h_{1}\right)\right)}^{2}}{2d}+\frac{{\left(x^{\prime }-\left(x+h_{1}\right)\right)}^{2}}{2d}+2z^{\prime }\sin\theta )]dx^{\prime }dz^{\prime }dzdz^{\prime \prime }dxdx^{\prime \prime }{|}^{2} \end{array} \end{array}$$

In Equation (4), we assumed:

- an uniform illumination of the target,
- an uniform illumination of the receiving antenna,
- a constant current distribution on the transmitting antenna.

The hypothesis of a constant current distribution stands for ultra-wide band antennas, and a quasi-uniform illumination can be assumed for ranges within the Fresnel region.

A PO, far-field expression for the RCS of a plate, $\sigma_{ff}$ can be found from Equation (4) when d→∞:

(6) $$\begin{array}{c} \begin{array}{cc} \sigma_{ff} & =\frac{4\pi \cos^{2}\theta }{\lambda^{2}}{\left|\int_{-\frac{b}{2}}^{\frac{b}{2}}\int_{-\frac{a}{2}}^{\frac{a}{2}}\exp\left(-2jkz^{\prime }\sin\theta \right)dx^{\prime }dz^{\prime }\right|}^{2}=\frac{4\pi a^{2}b^{2}\cos^{2}\theta }{\lambda^{2}}{\left[\frac{\sin(kb\sin\theta )}{kb\sin\theta }\right]}^{2} \end{array} \end{array}$$

and an analytical field-zone extrapolation factor *F* from Fresnel to far-field region can be defined:

(7) $$\begin{array}{c} F=\frac{\sigma_{Fr}}{\sigma_{ff}}=\frac{Q_{Fr_{plate}}}{a^{2}b^{2}{\left[\frac{\sin(kb\sin\theta )}{kb\sin\theta }\right]}^{2}}. \end{array}$$

When evaluating the six-integral expression in Equation (4), a computing time of several hours is needed on a customary computer, for a target of a size in the order of ten by ten minimal wavelengths, and for a fractional bandwidth in the order of unity. By using the Fresnel integrals, relation Equation (4) can be rewritten as a four-integral expression, Equation (A8). Since Fresnel integrals can be computed based on an asymptotic expansion, the computational time can be reduced form hours to minutes for oblique incidence, or even seconds for normal incidence; it should be noted that for an oblique incidence the expression of the pathlength difference is more complex than for normal incidence.

### 2.2. Case of a Complex Target Shape

In the Fresnel region, one should use either antennas of a size close to the target size, or antenna arrays, in order to completely illuminate the target. As an example, for measuring the RCS of a camping car of a typical size of 600 cm × 250 cm, a 1:10 scale model would make it possible to use customary horn antennas, provided that the frequency is multiplied by the same factor. A simplified target model, based on rectangular patches and slots, would serve as a reference for comparing measured results to analytical results. We assume the physical optics approximation, i.e., in the specular region, the surface current density on the surface of a flat target is twice the incident magnetic field, and it cancels in the shadow region. Thus, we solely analyze the RCS corresponding to the side of the camping-car, as no current density is considered on the other sides of the vehicle.

**Figure 3:**
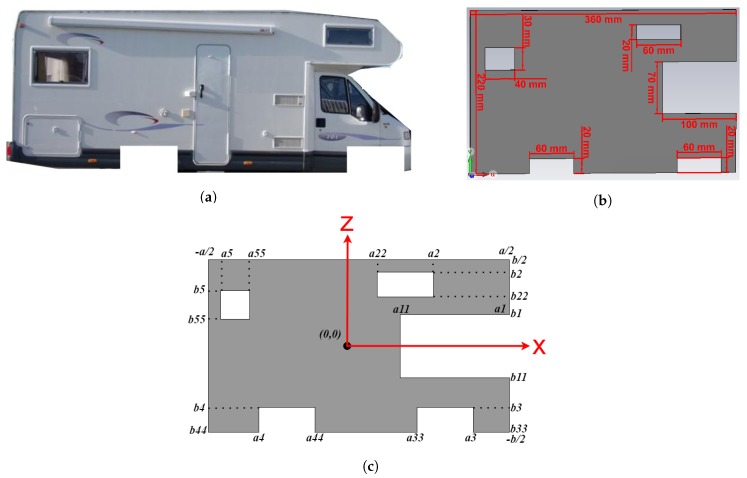
Camping car side as a complex target: (**a**) real target, (**b**) simulated model and (**c**) small scale model.

The magnetic field $H_{r_{c-c}}$ reflected by a simplified, small-scale model of a camping car side (Figure 3) at a distance *d* can be expressed as follows:

(8) $$\begin{array}{c} \begin{array}{cc} H_{r_{c-c}} & =H_{r}-H_{r_{1}}-H_{r_{2}}-H_{r_{3}}-H_{r_{4}}-H_{r_{5}}=j\frac{k\exp(-jkd)}{4\pi d}J_{S}\cos\theta Q_{FR_{c-c}}, \end{array} \end{array}$$

where

(9) $$\begin{array}{c} \begin{array}{cc} Q_{FR_{c-c}} & =\int_{-\frac{b}{2}}^{\frac{b}{2}}\int_{-\frac{a}{2}}^{\frac{a}{2}}\exp\left(-jk\Delta r\right)dx^{\prime }dz^{\prime }-\int_{b_{11}}^{b_{1}}\int_{a_{11}}^{a_{1}}\exp\left(-jk\Delta r_{1}\right)dx^{\prime }dz^{\prime }\\ \; & -\int_{b_{22}}^{b_{2}}\int_{a_{22}}^{a_{2}}\exp\left(-jk\Delta r_{2}\right)dx^{\prime }dz^{\prime }-\int_{b_{33}}^{b_{3}}\int_{a_{33}}^{a_{3}}\exp\left(-jk\Delta r_{3}\right)dx^{\prime }dz^{\prime }\\ \; & -\int_{b_{44}}^{b_{4}}\int_{a_{44}}^{a_{4}}\exp\left(-jk\Delta r_{4}\right)dx^{\prime }dz^{\prime }-\int_{b_{55}}^{b_{5}}\int_{a_{55}}^{a_{5}}\exp\left(-jk\Delta r_{5}\right)dx^{\prime }dz^{\prime }. \end{array} \end{array}$$

**Figure 4:**
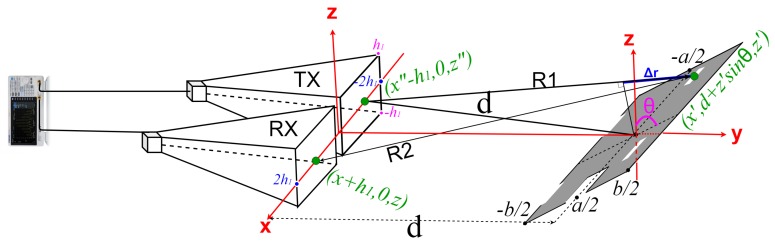
Measuring setup with horn antennas and a simplified, small scale model of camping car side.

In Equation (8), the magnetic field reflected by the target (Figure 3) is found by subtracting the magnetic field that would be reflected by five small rectangular plates $\left(H_{r_{1}},H_{r_{2}},H_{r_{3}},H_{r_{4}},H_{r_{5}}\right)$ of the same size as the slots corresponding to the non-reflective surface of the windows and wheels i.e., $\left(b_{1}-b_{11}\right)$ by $\left(a_{1}-a_{11}\right)$, $\left(b_{2}-b_{22}\right)$ by $\left(a_{2}-a_{22}\right)$, $\left(b_{3}-b_{33}\right)$ by $\left(a_{3}-a_{33}\right)$, $\left(b_{4}-b_{44}\right)$ by $\left(a_{44}-a_{4}\right)$ and $\left(b_{5}-b_{55}\right)$ by $\left(a_{55}-a_{5}\right)$ respectively. The path length differences $\Delta r_{i,(i\leq 5)}$ corresponding to each of the five small rectangular slots (Figure 4) are defined between any point in the area of interest and the reference point in the middle of the target.

Under the same assumptions as for deriving relation Equation (3) the RCS of the complex target at ranges in the Fresnel region is found as

(10) $$\begin{array}{c} \begin{array}{cc} \sigma_{Fr} & =4\pi d^{2}{\left|\frac{H_{r_{c-c}}}{H_{i}}\right|}^{2}=\frac{4\pi \cos^{2}\theta }{\lambda^{2}}{\left|\frac{1}{{\left(2h_{1}\right)}^{4}}\int_{-h_{1}}^{h_{1}}\int_{-h_{1}}^{h_{1}}\int_{-h_{1}}^{h_{1}}\int_{-h_{1}}^{h_{1}}Q_{FR_{c-c}}dxdzdx^{\prime \prime }dz^{\prime \prime }\right|}^{2}. \end{array} \end{array}$$

When d→∞, the PO, far-field RCS of the complex target $\sigma_{ff}$ is found. The field-zone extrapolation factor *F* from Fresnel to far-field zone is eventually found as

(11) $$\begin{array}{c} F=\frac{\sigma_{Fr}}{\sigma_{ff}}. \end{array}$$

As for the rectangular plate, the computing time can be reduced by expressing the RCS in terms of Fresnel integrals.

## 3. Measuring Setup for Validation and Results

**Figure 5:**
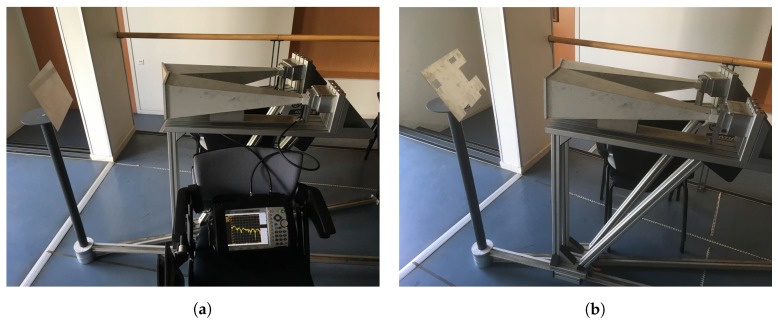
Measuring setup for validation for a rectangular plate (**a**) and a complex target (**b**).

**Figure 6:**
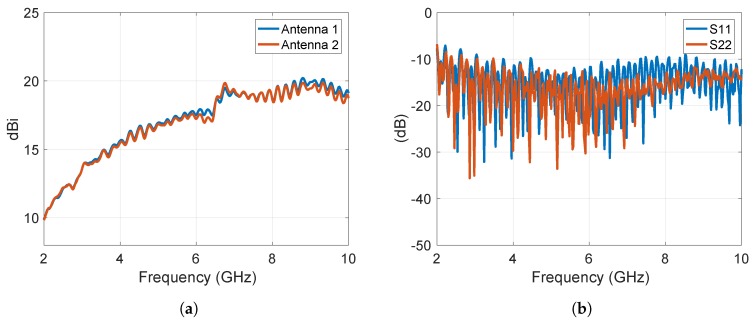
(**a**) Horn antenna gain and (**b**) magnitude of the input reflection coefficient as functions of frequency.

The method was validated by measurements at normal incidence $\left(\theta =0^{\circ }\right)$ and at incidence angles within the limits of the PO approximation $\left(\theta \leq 20^{\circ }\right)$. We choose as a target a rectangular plate of a size a=36 cm by b=22 cm (Figure 5a) and a small scale model of a camping car side (Figure 5b). With the notations in Section II, we chose $(b_{1}-b_{11})=7$ cm, $(a_{1}-a_{22})=10$ cm, $(b_{2}-b_{22})=2$ cm, $(a_{2}-a_{22})=6$ cm, $(b_{3}-b_{33})=2$ cm, $(a_{3}-a_{33})=6$ cm, $(b_{4}-b_{44})=2$ cm, $(a_{44}-a_{4})=6$, $(b_{5}-b_{55})=3$ cm, and $(a_{55}-a_{5})=4$ cm. The target was placed in a real multipath environment and the measurements were performed at frequencies between 2 and 10 GHz. A set of identical horn antennas of 15 cm × 15 cm in aperture size was used to measure the $S_{21}$ parameter at a set of Fresnel region antenna-to-target distances, i.e., *d* = 40 cm, 50 cm, 60 cm, 70 cm, 80 cm, 90 cm, and 100 cm. The gain of the horn antennas and the input reflection coefficient as functions of frequency are shown in Figure 6.

In order to find the Fresnel region RCS, the radar link equation was corrected with the field-zone extrapolation factor *F* from Equation (7) for the rectangular plate and Equation (11) for the complex target, by taking into account the impedance mismatch as well. All the measurements were performed in a multipath environment, and therefore the distance averaging technique was applied on the measured data [24,38] after the subtraction of the mutual coupling (CS) between the transmitting and receiving antenna:

(12) $$\begin{array}{c} \begin{array}{cc} \sigma_{ff}= & \frac{{\left(4\pi \right)}^{3}}{FG^{2}\lambda^{2}}\frac{|S_{21_{Fr}}^{total}-S_{21}^{coupling}{|}^{2}}{|1-S_{22}{|}^{2}}\frac{R_{0}}{R_{a}\left(1-\right|S_{11}{|}^{2})}. \end{array} \end{array}$$

In Equation (12), $R_{0}$ is the normalizing impedance (50 ohm), $R_{a}$ is the radiation resistance of the receiving antenna, *G* is the gain of each antenna, $S_{21}^{coupling}$ is the $S_{21}$ parameter measured without the target and $S_{21_{Fr}}^{total}$ is a normalized transfer function computed as an average figure over the set of distances $d_{n}$ [24]:

(13) $$\begin{array}{c} S_{21_{Fr}}^{total}=\frac{1}{N}\sum_{n=1}^{N}(\frac{d_{n}}{d_{0}}{)}^{2}\left|S_{21,n}\exp\left(2jkd_{n}\right)\right| \end{array}$$

with $d_{0}$ a reference distance usually set at 1 m.

Measurements were performed on a set of 401 frequencies, equally spaced between 2 and 10 GHz.

**Figure 7:**
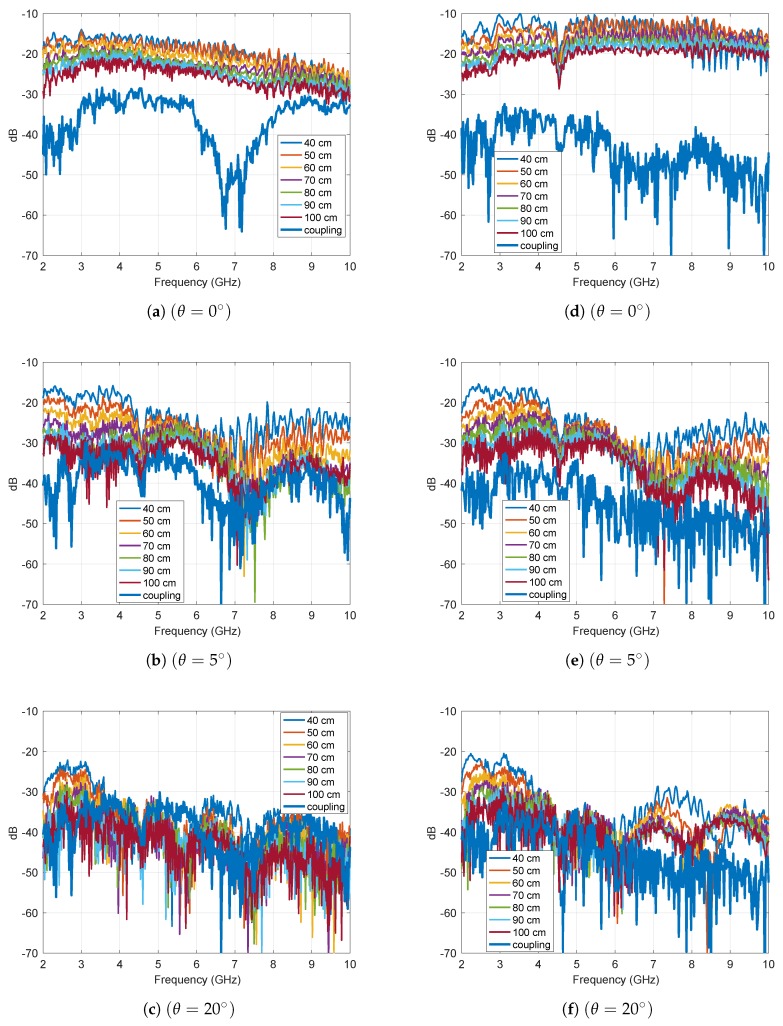
Frequency domain representation of the magnitude of the $S_{21}$ parameters measured for a rectangular plate at $\theta =0^{\circ }$ (**a**), $\theta =5^{\circ }$ (**b**), and $\theta =20^{\circ }$ (**c**), and for a small scale model of a camping car side at $\theta =0^{\circ }$ (**d**), $\theta =5^{\circ }$ (**e**), and $\theta =20^{\circ }$ (**f**).

Figure 7 displays the frequency domain representation of the magnitude of $S_{21}$ measured for a rectangular plate and for a small scale model of a camping car side at $\theta =0^{\circ }$, $5^{\circ }$ and $20^{\circ }$. The mentioned coupling in the figures refers to $S_{21}$ measured without target. Figure 7 shows that as the angle of incidence increased, the magnitude of $S_{21}$ decreased and became comparable to the level of the mutual coupling. In this case, by subtracting the coupling between transmitter and receiver, the accuracy on RCS evaluation in the Fresnel region may improve.

**Figure 8:**
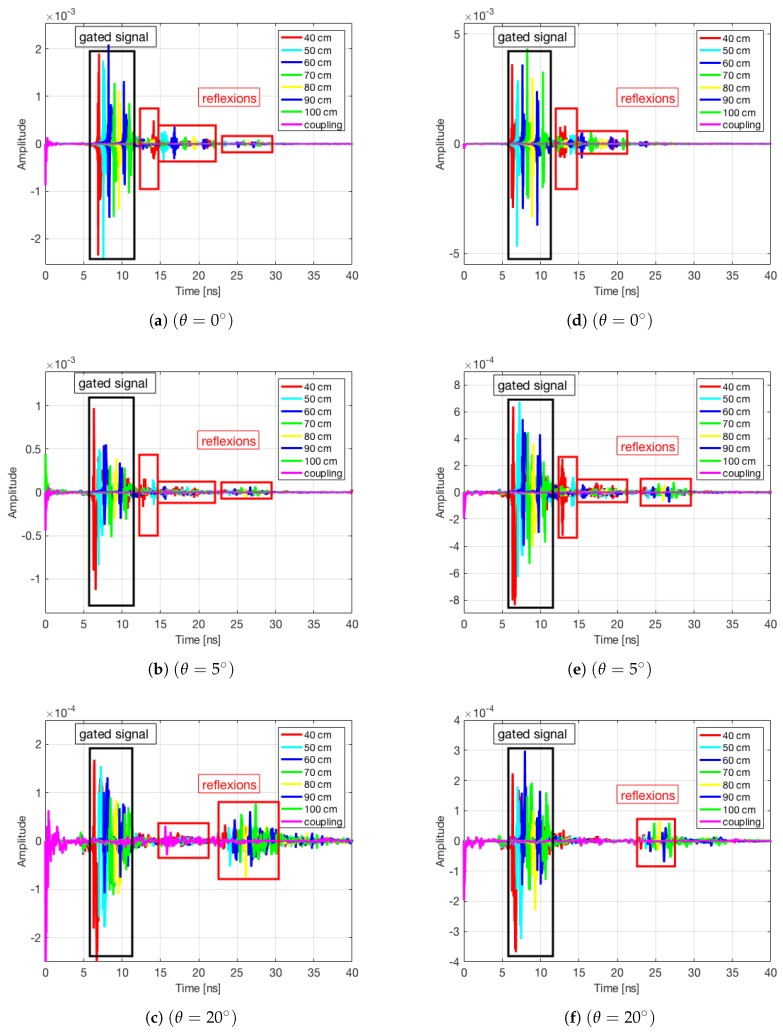
Time domain representation of the $S_{21}$ parameters measured for a rectangular plate at $\theta =0^{\circ }$ (**a**), $\theta =5^{\circ }$ (**b**), and $\theta =20^{\circ }$ (**c**), and for a small scale model of a camping car side at $\theta =0^{\circ }$ (**d**), $\theta =5^{\circ }$ (**e**), and $\theta =20^{\circ }$ (**f**).

As an alternative to the coupling subtraction one can apply the time-gating technique. Figure 8 displays the inverse Fourier transform of $S_{21}$ measured for a rectangular plate and for a small scale model of a camping car side at $\theta =0^{\circ }$, $5^{\circ }$ and $20^{\circ }$. It can be noted that at high incidence angles ($\theta =20^{\circ }$), the amplitude of the wave reflected by the target was comparable to the amplitude of the late waves reflected by different obstacles in the environment. In this case, a distance averaging after a time gating performed on the impulse response between 5 ns and 12 ns will remove the effects of the late reflexions.

**Figure 9:**
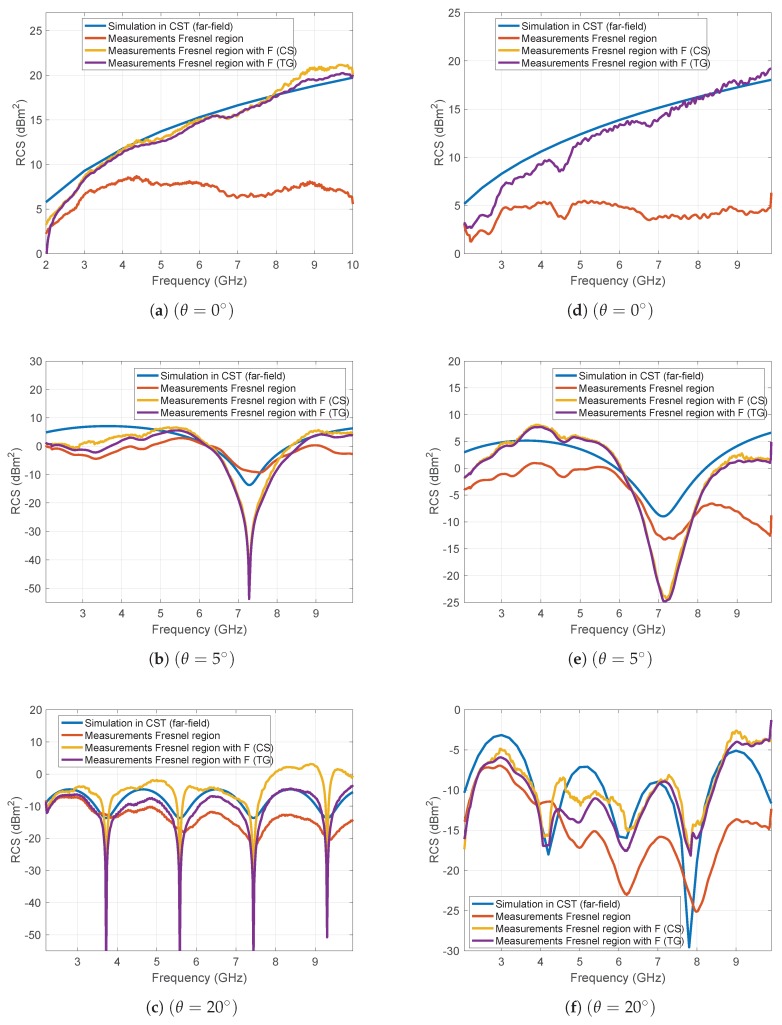
A comparison between the RCS simulated in CST, the RCS measured at Fresnel region distances and the RCS measured at Fresnel region distances corrected with the field-zone extrapolation factor F after coupling subtraction (CS) or time gating (TG) for a rectangular plate at $\theta =0^{\circ }$ (**a**), $\theta =5^{\circ }$ (**b**), and $\theta =20^{\circ }$ (**c**), and for a small scale model of a camping car side at $\theta =0^{\circ }$ (**d**), $\theta =5^{\circ }$ (**e**), and $\theta =20^{\circ }$ (**f**).

Figure 9 displays a comparison between RCS figures simulated in CST, and measured at Fresnel region distances, with and without correcting for the field zone with the field-zone extrapolation factor *F* (after coupling subtraction or time gating). More specifically, in Figure 9a–c we show the RCS results for the rectangular plate at normal incidence $\left(\theta =0^{\circ }\right)$ and non-normal incidence angles of 5 and 20 degrees, respectively. Figure 9d–f show RCS results for the complex target i.e., a small scale model of a camping car side at normal incidence $\left(\theta =0^{\circ }\right)$ and at incidence angles of 5 and 20 degrees, respectively.

At incidence angles where the amplitude of the direct reflexions is comparable to the amplitude of the late reflexions, the subtraction of the coupling between transmitter and receiver will lead to less accurate RCS results; conversely, the utilization of the time gating prior to the distance averaging will improve the accuracy for evaluating RCS figures corrected with *F*. However, at normal incidence, where the amplitude of the late reflexions is several times smaller than target reflexion and the signal-to-coupling ratio is high, both techniques show same accuracy.

## 4. Conclusions

When extracting the radar cross section from Fresnel zone measurements, discrepancies of up to 15 dB can be noted, compared to far-field results. We therefore defined a field-zone extrapolation factor to be applied on the RCS figures measured in the Fresnel region. By approximating the extrapolation factor in terms of Fresnel integrals the computing time can be dramatically reduced (typically from hours to minutes), compared to exact field-zone extrapolation approaches. It should be emphasized that our technique requires a simple, low-cost setup consisting of two identical horn antennas and a vector network analyzer. Moreover, we showed that our method can be used in a perturbed, multipath environment; a data processing approach including distance averaging, time-gating, and antenna coupling subtraction makes it possible to measure the radar cross section of large targets e.g., aircrafts, vessels, or other large vehicles, without needing an anechoic chamber. There is a good agreement between the RCS figures extracted with our method from measurements in the Fresnel zone, and simulations. In order to validate our approach on a complex target, we used a simplified model consisting of rectangular patches and slots; the far-field RCS of such a model can further be evaluated analytically for comparison purposes. Further work will focus on increasing the incidence angle and on taking into account the effects of the diffraction.

## Appendix A

### Appendix A.1

The path length difference Δr can be expressed as

(A1) $$\begin{array}{c} \Delta r=R_{1}+R_{2}-2d \end{array}$$

where

(A2) $$\begin{array}{c} R_{1}=\sqrt{{\left(x^{\prime }-\left(x^{\prime \prime }-h_{1}\right)\right)}^{2}+{\left(d+z^{\prime }\sin\theta \right)}^{2}+{\left(z^{\prime \prime }-z^{\prime }\right)}^{2}}, \end{array}$$

(A3) $$\begin{array}{c} R_{2}=\sqrt{{\left(x^{\prime }-\left(x+h_{1}\right)\right)}^{2}+{\left(d+z^{\prime }\sin\theta \right)}^{2}+{\left(z-z^{\prime }\right)}^{2}}. \end{array}$$

By assuming

(A4) $$\begin{array}{c} d^{2}\gg {\left(\frac{a}{2}+2h_{1}\right)}^{2}+{\left(h_{1}+\frac{b}{2}\right)}^{2}, \end{array}$$

(A1) becomes

(A5) $$\begin{array}{c} \begin{array}{cc} \Delta r= & \frac{{\left(z-z^{\prime }\right)}^{2}}{2d}+\frac{{\left(z^{\prime \prime }-z^{\prime }\right)}^{2}}{2d}+\frac{{\left(x^{\prime }-\left(x^{\prime \prime }-h_{1}\right)\right)}^{2}}{2d}+\frac{{\left(x^{\prime }-\left(x+h_{1}\right)\right)}^{2}}{2d}+2z^{\prime }\sin\theta . \end{array} \end{array}$$

### Appendix A.2

$Q_{Fr_{plate}}$ can be rewritten as

(A6) $$\begin{array}{c} \begin{array}{cc} Q_{Fr_{plate}} & =\frac{1}{{\left(2h_{1}\right)}^{4}}\int_{-h_{1}}^{h_{1}}\int_{-h_{1}}^{h_{1}}\int_{-\frac{b}{2}}^{\frac{b}{2}}\exp\left[-jk\left(\frac{{\left(z-z^{\prime }\right)}^{2}}{2d}+\frac{{\left(z^{\prime \prime }-z^{\prime }\right)}^{2}}{2d}+\frac{{\left(z^{\prime }\right)}^{2}}{2d}+2z^{\prime }\sin\theta \right)\right]dz^{\prime }dzdz^{\prime \prime }\\ \; & \times \int_{-h_{1}}^{h_{1}}\int_{-h_{1}}^{h_{1}}\int_{-\frac{a}{2}}^{\frac{a}{2}}\exp\left[-jk\left(\frac{{\left(x^{\prime }-\left(x^{\prime \prime }-h_{1}\right)\right)}^{2}}{2d}+\frac{{\left(x^{\prime }-\left(x+h_{1}\right)\right)}^{2}}{2d}\right)\right]dx^{\prime }dxdx^{\prime \prime }. \end{array} \end{array}$$

By using the Fresnel integral

(A7) $$\begin{array}{c} f\left(w\right)=\int_{w}^{\infty }\exp\left(-ju^{2}\right), \end{array}$$

$Q_{Fr_{plate}}$ becomes

(A8) $$\begin{array}{c} \begin{array}{cc} \; & Q_{Fr_{plate}}=\frac{1}{{\left(2h_{1}\right)}^{4}}\int_{-h_{1}}^{h_{1}}\int_{-h_{1}}^{h_{1}}\int_{-\frac{b}{2}}^{\frac{b}{2}}\exp\left[-jk\left(\frac{{\left(z-z^{\prime }\right)}^{2}}{2d}+\frac{{\left(z^{\prime \prime }-z^{\prime }\right)}^{2}}{2d}+\frac{{\left(z^{\prime }\right)}^{2}}{2d}+2z^{\prime }\sin\theta \right)\right]dz^{\prime }dzdz^{\prime \prime }\\ \; & \times \frac{2d}{k}\int_{-\frac{a}{2}}^{\frac{a}{2}}\left\{\left[f\left(x^{\prime }\sqrt{\frac{k}{2d}}\right)-f\left(\left(x^{\prime }-2h_{1}\right)\sqrt{\frac{k}{2d}}\right)\right]\left[f\left(\left(x^{\prime }+2h_{1}\right)\sqrt{\frac{k}{2d}}\right)-f\left(x^{\prime }\sqrt{\frac{k}{2d}}\right)\right]dx^{\prime }\right\}. \end{array} \end{array}$$
